# PET microplastics alter the transcriptome profile and oxidative stress markers in the liver of immature piglets: an in vivo study

**DOI:** 10.1007/s00204-025-04151-8

**Published:** 2025-08-17

**Authors:** Monika Golubska, Aleksandra Kurzyńska, Karol Mierzejewski, Ismena Gałęcka, Jarosław Całka, Iwona Bogacka

**Affiliations:** 1 Department of Animal Anatomy and Physiology, Faculty of Biology and Biotechnology, University of Warmia and Mazury in Olsztyn, Olsztyn, Poland; 2 Department of Epizootiology, Faculty of Veterinary Medicine, University of Warmia and Mazury in Olsztyn, Olsztyn, Poland; 3 Department of Clinical Physiology, Faculty of Veterinary Medicine, University of Warmia and Mazury in Olsztyn, Olsztyn, Poland

**Keywords:** Pig, Microplastics, Polyethylene terephthalate, Liver, RNA-Seq

## Abstract

The increasing global contamination with microplastics (MPs) poses a significant threat to human and animal health. Recent studies suggest that exposure to microplastics contributes to various detrimental hepatic effects, including oxidative stress and metabolic dysregulation. The aim of the present study was to investigate the global liver transcriptome, oxidative stress and selected liver function markers in immature piglets (*n* = 15) exposed to polyethylene terephthalate (PET) MPs for 4 weeks. The animals were divided into three groups: a low-dose MPs exposure group (0.1 g PET MPs/day), a high-dose MPs exposure group (1 g PET MPs/day), and a control group that did not receive MPs. The transcriptome profile of the liver was assessed using RNA-Seq. In addition, markers of oxidative stress (catalase, superoxide dismutase, glutathione peroxidase, glutathione transferase, and malondialdehyde) were determined using specific enzymatic assays, and the levels of selected liver function markers (bilirubin, collagen IV, alanine transaminase and aspartate aminotransferase) were measured by ELISA. The results showed that exposure to MPs, especially at a high dose, significantly altered the hepatic transcriptome profile. A low dose of PET MPs changed the expression of 5 genes, while a high dose affected the expression of 24 genes. The differentially expressed genes were associated with several biological processes such as cholesterol metabolism, transferase activity, and oxidation. Moreover, consumption of MPs resulted in increased catalase activity and decreased activity of superoxide dismutase and glutathione peroxidase in the liver. We also observed an increase in bilirubin and a decrease in collagen type IV, alanine aminotransferase, and aspartate aminotransferase content in the liver. These results suggest that PET MPs ingestion may disrupt systemic homeostasis and contribute to liver dysfunction.

**Supplementary Information:**

The online version contains supplementary material available at 10.1007/s00204-025-04151-8.

## Introduction

Plastic and microplastic pollution are a growing public problem. It is estimated that 368 million tons of plastic were produced worldwide in 2019 (PlasticsEurope 2020). Taking into account population growth and waste habits, this production is expected to increase (PlasticsEurope 2020). The main focus should be on microplastics (MPs). These are plastic particles that are smaller than 5 mm and can accumulate in various organs due to their small size (Deng et al. 2017). Humans are exposed to microplastics in many ways, including through inhalation and skin contact, but food consumption remains the main source of exposure (Prata et al. 2020). It should be emphasized that microplastic particles have been found in various foods such as water, seafood, meat products, fruits, and vegetables or sugar (Kedzierski et al. 2020; Oliveri Conti et al. 2020; Toussaint et al. 2019; Yee et al. 2021).

In Europe, the primary polymers produced include polyethylene (PE), polypropylene (PP), polyvinyl chloride (PVC), polyurethane (PUR), polyethylene terephthalate (PET), and polystyrene (PS) (PlasticsEurope 2020). Among these, PET is extensively utilized in the food packaging industry for the production of bottles/containers for beverages such as water, carbonated soft drinks, or other drinks/juices (Nisticò, 2020). These polymer particles have been found in human blood and feces (Leslie et al. 2022; Schwabl et al. 2019; Yan et al. 2022). The prevalence of PET MPs in human feces varies across life stages. It is estimated that the concentration of PET particles in the feces of infants is much higher (up to 10–20 times) than in adults, suggesting that infants are exposed to greater doses of MPs (Zhang et al. 2021).

A growing number of studies have focused on the potential effects of MPs on human health (Fournier et al. 2021; Lu et al. 2019; Yee et al. 2021). However, most evidence for the harmfulness of plastic comes from research on aquatic organisms, rodents or in vitro models, making it difficult to translate these findings to human health (Senathirajah et al. 2021). Therefore, it is justified to use larger mammals as model organisms, such as pigs, which have many anatomical and physiological similarities to humans (Lunney et al. 2021). Since the main source of exposure to microplastics is food contact, it seems reasonable to study the digestive system and its glands, such as the liver, in particular. The accumulation of microplastic particles has been confirmed in liver tissue samples from patients with liver cirrhosis, but not in healthy patients (Horvatits et al. 2022). Exposure to microplastics in vivo in mice, zebrafish and marine medaka resulted in a number of liver disorders, including disturbances in lipid and energy metabolism, oxidative stress, and inflammatory responses (Deng et al. 2017; Limonta et al. 2019; Ye et al. 2021). MPs’ exposure also results in changes at transcriptomic level (Patra et al. 2022; Tang 2025). Studies on PS in zebrafish gills and liver, and in goldfish liver, have indicated changes in the expression of several antioxidant-related genes like *CAT, SOD*, and *GSTR* (Abarghouei et al. 2021; Xu et al. 2021) as well as genes involved in inflammatory responses (e.g., *TNFα, IL-6, CXCL11*) in rat astrocytes, mouse bone marrow and zebrafish gills and liver (Marcellus et al. 2024; Sun et al. 2021; Xu et al. 2021). PVC MPs’ exposure has been shown to activate the PI3K/Akt pathway related to apoptosis and oxidative stress in mice liver and MAPK and TGF-beta signaling pathway in human bronchial epithelial cell line (Chen et al. 2022; Liu et al. 2024). Exposure to PE in larval zebrafish and polystyrene in zebrafish liver resulted in changes in levels of glucose and lipid metabolism-related genes (e.g., *PPARα*, *FAS*, *PPAR-γ*, *APO, GK, PEPCKc, HK1)* (Luo et al. 2021; Zhao et al. 2021). Moreover, PE MPs altered the expression of oxidative stress-related genes (*L-FABP, CAT, SOD3)* in zebrafish larvae and sheepshead minnow larvae (Choi et al. 2018; Kurchaba et al. 2020).

Our previous studies have demonstrated that MPs undergo extracellular vesicles-mediated translocation, with their miRNA cargo modulating the expression of genes associated with metabolic dysregulation and carcinogenesis, including hepatocellular carcinoma (HCC) (Mierzejewski et al. 2023). Based on the extensive use of PET, the potential adverse health effects associated with MPs, existing literature data involving other animal species, and our previous studies, we hypothesize that oral exposure to PET may affect liver biology. Therefore, the aim of the present studies was to investigate the effect of oral exposure to two doses of PET MPs (0.1 g/day and 1 g/day) for 4 weeks on the liver transcriptome profile, oxidative stress markers, and selected liver function markers in immature gilts.

## Materials and methods

### Animals

The study was conducted in accordance with the requirements of the European Union Directive on the ethical use of experimental animals (EU Directive 2010/63/EU on animal experimentation). The experimental protocols were approved by the Local Ethics Committee of the University of Warmia and Mazury in Olsztyn (Decision No. 10/2020 of February 26, 2020).

The animals were prepared as previously described (Gałęcka et al. 2024). In brief, the experiment was conducted on 8-week-old (Pietrain x Duroc) immature gilts (*n* = 15) with an approximate body weight of 20 kg. The animals were kept in the breeding pens under standard laboratory conditions with unlimited availability of fresh water (ad libitum) and an age-appropriate feed mixture. The temperature in the breeding enclosure was kept at 20–22 °C and the humidity was 55–60%. All plastic materials were removed from the animals’ environment. The watering troughs and feeders in the pens were made of stainless steel. The environment of the farm where the gilts were reared before the experiment was also made of stainless steel. Components to support the animals’ well-being were made of wood or bedding. Three experimental groups were formed (5 animals per group): 1) control group (CTR)—it received an empty gelatin capsule; 2) low-dose experimental group (LD)—it received a low dose of PET MPs (0.1 g/pig/day in gelatin capsules); 3) high-dose experimental group (HD)—it received a high dose of PET MPs (1 g/pig/day in gelatin capsules). One hour before morning feeding, gelatin capsules were administered to the gilts *per os*.

The MPs used in the experiment were a semi-crystalline polyethylene terephthalate powder (cat. no. ES306031/1Goodfellow Cambridge Ltd., Huntingdon, England), which was characterized using dynamic light scattering and microscopy to assess particle size and morphology as previously described (Gałęcka et al. 2025). Particle size distribution was determined with a Mastersizer 2000 (Malvern Instruments Ltd., UK) using the dry method with Scirocco 2000 device. Particles ranged from 7.6 to 416.9 µm, with a dominant size of 158.5 µm. Analysis showed that 10% of particles had diameters ≤ 51.6 µm, 50% ≤ 124.6 µm, and 90% ≤ 237.0 µm, with a mean diameter of 135.6 µm. Microscopic observations using a Zeiss Axio Imager.M2 (Zeiss, Oberkochen, Germany) and Phenom ProX G6 scanning microscope (ThermoFisher Scientific, Waltham, MA, USA) revealed that particles varied in shape and had rough, uneven surfaces. The use of MPs particles of different sizes is well justified, as different sizes of MPs (mostly less than 300 μm) have been identified in human feces (Yan et al. 2022). Moreover, it has been previously shown that the use of a mixture of different sizes of MPs leads to negative effects on the physiology of the digestive system (Djouina et al. 2022). The plastic particles had different shapes: spherical, fibrous, irregular and had both sharp and rounded edges. The applied doses of microplastics were determined based on the estimated humans’ consumption of microplastics in the literature, which is estimated at a weekly level between 0.1 to 5 g, although individual microplastic consumption can vary depending on a combination of many factors such as age, diet, lifestyle, or living environment (Senathirajah et al. 2021). Taking into account a number of factors that may influence individual consumption of MPs, it is difficult to select a single dose of microplastic for in vivo testing. The low dose was also determined based on toxicology studies with mice (Deng et al. 2017) and adjusted to the weight of pigs, while the high dose was ten times higher to simulate an overload scenario (Yang et al. 2019).

The experiment lasted 4 weeks, after which the pigs were pre-medicated atropine (0.05 mg/kg *i.m*., Polfa, Warsaw, Poland), followed by induction with xylazine (3 mg/kg *i.m*., Vet-Agro, Lublin, Poland) and ketamine (6 mg/kg *i.m.,* Vetoquinol Biowet, Gorzów Wlkp., Poland), and then euthanized with an overdose of sodium pentobarbital (0.6 ml/kg, *iv*., Biowet, Puławy, Poland). After the cessation of biological functions was confirmed, the abdominal cavity was opened and tissue fragments were removed from the left lobe of the liver. The tissue fragments were then cleaned and rinsed with ice-cold PBS, then immediately frozen in liquid nitrogen and stored at −80 °C for further analysis.

### RNA isolation, library preparation, and sequencing procedure

Total RNA was isolated from 15 samples (5 explants from the CTR group, 5 explants from the LD group, 5 explants from the HD group) using the “RNeasy Mini Kit” (Qiagen, Hilden, Germany) in accordance with the manufacturer’s instructions. The concentration and purity of the isolated RNA were measured using a Tecan Infinite M200 plate reader (Tecan Group Ltd., Switzerland). The RNA Integrity Number (RIN) for each sample was estimated with an Agilent Bioanalyzer 2100 (Agilent Technology, USA) and the Agilent RNA 6000 Nano Kit (Agilent Technology, USA). All samples exhibited RIN values above 8. Poly(A) mRNAs were purified through two rounds of purification using oligo(dT) magnetic beads. The poly(A) RNA was then fragmented using a divalent cation buffer. Transcription of the RNA into cDNA was performed using poly(DT) oligonucleotides. The cDNA was then subjected to 3'adenylation and adapter ligation. Reverse transcription during library construction was strand-specific. Finally, the libraries were pooled and sequenced. Quality control and quantification of the sequencing libraries were performed using the Agilent Technologies 2100 Bioanalyzer with the High Sensitivity DNA Chip. Paired-end sequencing was conducted using the Illumina NovaSeq 6000 system (LC Science, Houston, TX, USA).

### Bioinformatics analysis

FastQC (v0.10.1) was used for assessing sequence quality. After filtering out low-quality reads, the remaining 150 bp paired-end sequences were assembled and mapped to the *Sus scrofa* genome (v107) using HISAT2 (v2.0). The mapped reads for each sample were assembled using StringTie (v1.3.4). All transcriptomes were then merged using Perl scripts and GffCompare to create a comprehensive transcriptome. After creating the final transcriptome, StringTie and edgeR were employed to estimate transcript expression levels. StringTie calculated the expression of mRNAs in fragments per kilobase of transcript per million (FPKM). Differentially expressed genes (DEGs) were identified based on a log_2_(fold change) > 1 or log_2_(fold change) < −1, and statistical significance was determined with a p-adjusted < 0.05 using the R package edgeR.

### Oxidative stress markers analysis

Oxidative stress markers’ activities were measured using commercial enzymatic kits from Cayman Chemical: Superoxide Dismutase Assay Kit (SOD, 706,002), Catalase Assay Kit (CAT, 707,002), Glutathione Peroxidase Assay Kit (GPx, 703,102), Glutathione S-Transferase Assay Kit (GST, 703,302), and TBARS (TCA Method) Assay Kit (700,870) for malondialdehyde (MDA, a lipid peroxidation product). The activities were analyzed in liver tissue samples, each weighing approximately 25 mg, following the manufacturer’s instructions. With the exception of MDA, all activities were normalized to the protein concentration, which was determined using the BCA Protein Assay (ThermoFisher, USA). Absorbance values were measured with the Infinite M200 Pro Reader, using Tecan i-control software (Tecan, Switzerland).

### Enzyme-linked immunosorbent assay (ELISA)

The protein concentrations of total bilirubin (Porcine Total bilirubin ELISA Kit, Cat. No EA0061Po, BT LAB, China), collagen type IV (Porcine Collagen Type 4 ELISA Kit, Cat. No E0020Po, BT LAB, China), alanine aminotransferase (ALT, Porcine Alanine Aminotransferase ELISA Kit, Cat. No E0150Po, BT LAB, China), and aspartate aminotransferase (AST, Porcine Aspartate Aminotransferase, cytoplasmic ELISA Kit, Cat. No E0529Po, BT LAB, China) in tissue homogenates were determined using a commercial ELISA kit specific for pigs, following the manufacturer’s protocol as previously described. The standard curve ranges for each protein were as follows: bilirubin (50–3200 ng/ml), collagen type IV (3–900 ng/ml), ALT (2.5–160 ng/ml), AST (0.1–40 ng/ml). Absorbance measurements were taken at a wavelength of 450 nm using the Infinite M200 Pro Reader with Tecan i-control software (Tecan, Switzerland). The intra- and inter-assay coefficients of variation for the ELISA assays of the selected proteins were: 10% and 12% for bilirubin and 8% and 10% for collagen type IV, AST and ALT.

### Validation of the obtained results by real-time PCR

Real-time PCR analysis was performed using the Power SYBR Green PCR Master Mix (Applied Biosystems, USA) and AriaMx Real-Time PCR System (Agilent Technology, USA), as described previously (Mierzejewski et al. 2021). Primer sequences for reference and target genes (*GAPDH, ADIPOQ, APOA4, SULT1B1, GSTM3*) were designed using Primer Express Software 3 (Applied Biosystems, USA). The abundance of the tested mRNAs was estimated using standard curves prepared by a serial dilution of a known quantity of total RNA. The constitutively expressed *GAPDH* gene was used as reference gene. Gene expression data were normalized to *GAPDH* expression by dividing the expression values of the tested gene by the reference gene and presented as arbitrary units. Real-time PCR results were analyzed with Statistica software (Statsoft Inc. Tulsa, USA) using Student’s *t* test and expressed as means ± SEM. The results were considered statistically significant at p ⩽ 0.05.

### Statistical analysis

Statistical analysis of mRNA abundance, protein concentration, and oxidative stress markers in the experimental tissue was performed using Statistica (version 13, StatSoft Inc., Tulsa, OK, USA). The Shapiro–Wilk test was used to confirm the normality of the distribution in each tested group. The Brown–Forsythe test was used to check the equality of variances between the tested groups. The effect of the tested doses of PET MPs compared to the control was examined using the Student’s *t* test. The results were considered statistically significant at *p* ⩽ 0.05.

## Results

### Statistics of RNA sequencing

RNA sequencing data were created for 15 cDNA libraries, including 5 for the control group, 5 for the LD group, and 5 for HD group. The analysis produced 632,806,112 raw paired-end reads in total, with an average 42,187,074 per sample and a Q20 value that was on average 98.9%. The short reads, low-quality sequences, and ambiguous nucleotides were removed from the raw reads, leaving 603,436,666 valid reads, and an average 40,229,111 reads per sample. These reads were further mapped to the 107 version of the pig genome with a unique mapped average rate of 92.91%. The analysis of the distribution of mapped reads to gene structures indicated that average 96.81% reads were mapped to coding sequences, 2.16% to introns and 1.03% to intergenic regions.

### Impact of a low dose of PET microplastics on differentially expressed genes (DEGs)

**Fig. 1:**
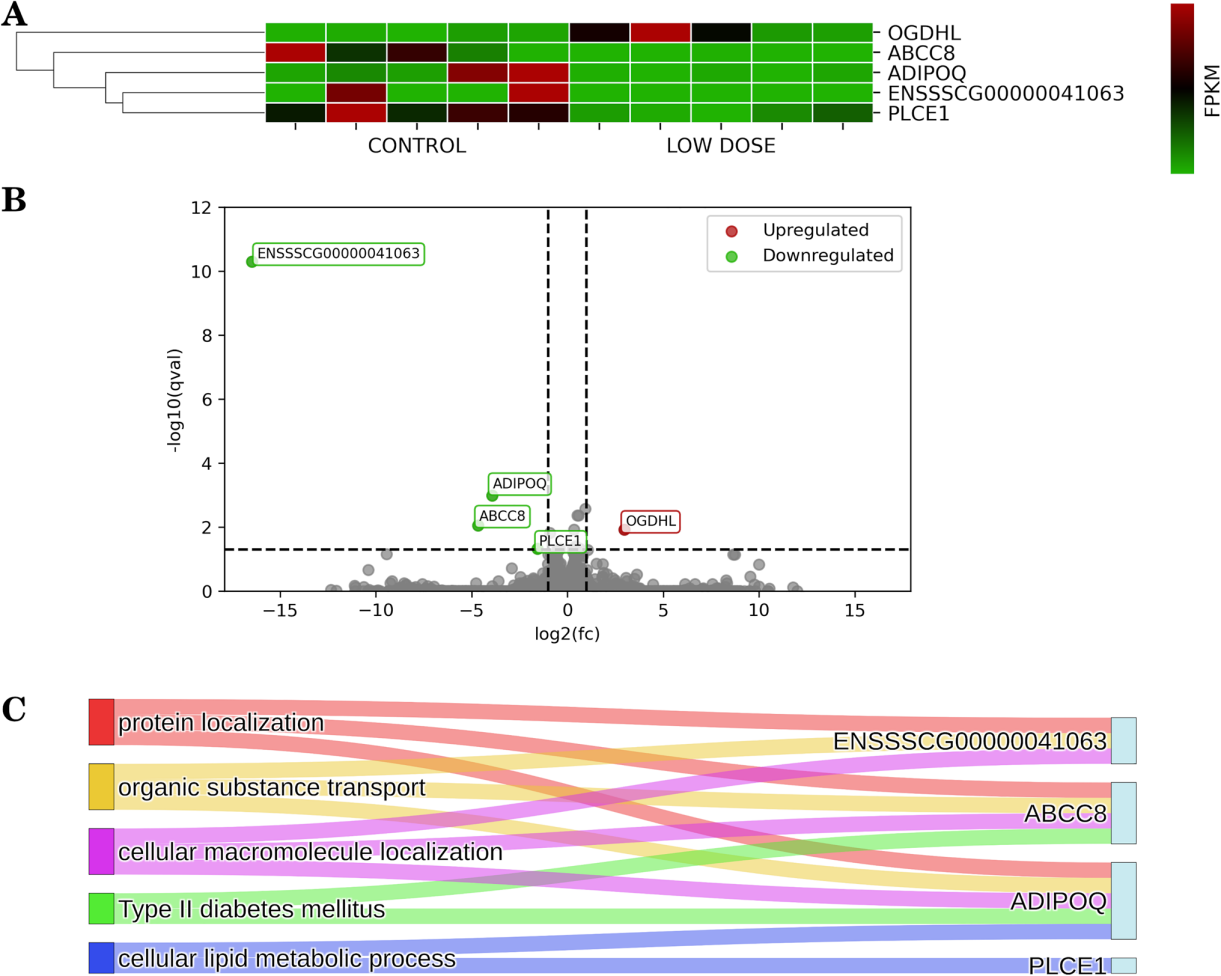
**A** Heatmap illustrating the expression of differentially expressed genes. The plot shows FPKM (Fragments Per Kilobase of transcript per Million mapped reads) values for the low dose condition relative to the control, along with hierarchical clustering of genes represented by a dendrogram. **B** Volcano plot depicting all expressed genes, with significant genes highlighted and labeled. The analysis compares the low dose condition to the control. Green dots indicate downregulated genes relative to the control, while red dots indicate upregulated genes. Genes with no significant changes are represented as gray dots. **C** Sankey diagram representing the associations between differentially expressed genes (between low dose and control conditions) and their corresponding GO (Gene Ontology) terms and KEGG (Kyoto Encyclopedia of Genes and Genomes) pathways

Following the consumption of a low dose of microplastics, we identified five DEGs, including one that was upregulated and four that were downregulated (Fig. 1). Gene Ontology (GO) analysis revealed that DEGs were associated with 5 molecular function (MF) terms, 212 biological process (BP) terms, and 4 cellular component (CC) terms. In addition, DEGs were associated with six Reactome (REAC) pathways and one Kyoto Encyclopedia of Genes and Genomes (KEGG) pathway. The identified genes were involved in processes such as protein localization (*ENSSSCG00000041063, ADIPOQ, ABCC8*), enzyme-linked receptor protein signaling pathway (*PLCE1, ADIPOQ*), organic substance transport (*ENSSSCG00000041063, ADIPOQ, ABCC8*), and type II diabetes mellitus (*ADIPOQ, ABCC8*). Detailed results of DEGs and GO analyses can be found in part A of Supplemental Table 1 and Supplemental Table 2.

### Impact of a high dose of PET microplastics on differentially expressed genes (DEGs)

**Fig. 2:**
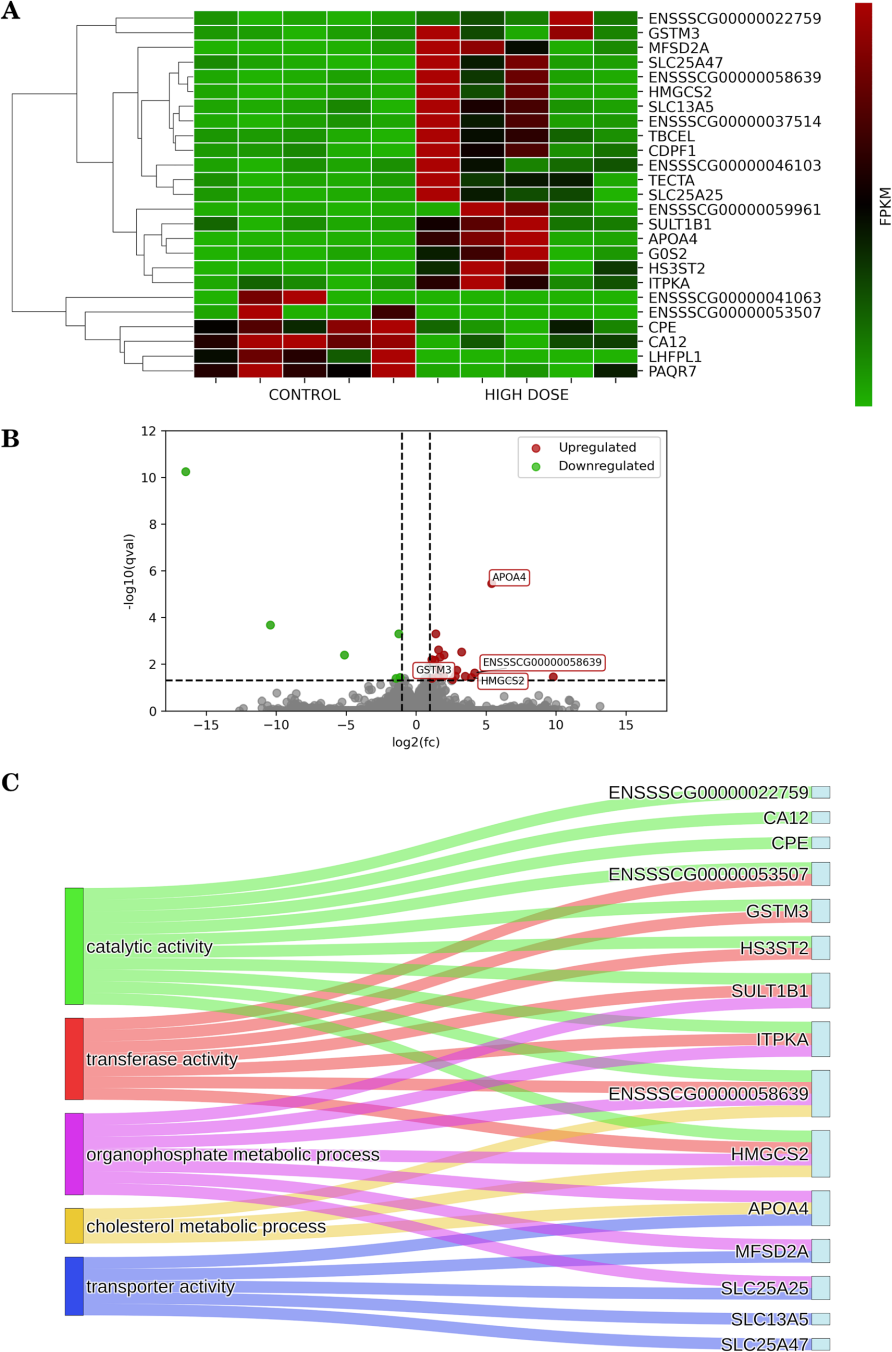
**A** Heatmap illustrating the expression of differentially regulated protein-coding genes. The plot shows FPKM (Fragments Per Kilobase of transcript per Million mapped reads) values for the high-dose condition relative to the control, along with hierarchical clustering of genes represented by a dendrogram. **B** Volcano plot depicting all expressed genes, with significant genes highlighted and labeled. The analysis compares the high dose condition to the control. Green dots indicate downregulated genes relative to the control, while red dots indicate upregulated genes. Genes with no significant changes are represented as gray dots. **C** Sankey diagram representing the associations between differentially expressed genes (between high-dose and control conditions) and their corresponding GO (Gene Ontology) terms and KEGG (Kyoto Encyclopedia of Genes and Genomes) pathways

Following the consumption of a high dose of microplastics, we detected 24 DEGs, comprising 18 that were upregulated and 6 that were downregulated (Fig. 2). GO analysis categorized these DEGs to 62 molecular function (MF) terms, 176 biological process (BP) terms, 10 KEGG pathways, and 15 Reactome (REAC) pathways. Notably, among the identified processes were those related to cholesterol metabolic processes (*APOA4, ENSSSCG00000058639, HMGCS2*), transferase activity (*ENSSSCG00000053507, SULT1B1, ENSSSCG00000058639, GSTM3, HS3ST2, HMGCS2, ITPKA*), and catalytic activity (*ENSSSCG00000053507, ENSSSCG00000022759, SULT1B1, ENSSSCG00000058639, GSTM3, HS3ST2, HMGCS2, CA12, CPE, ITPKA*). Detailed results of DEGs and GO analyses can be found in part B of Supplemental Table 1 and Supplemental Table 2.

### Impact of PET microplastics on oxidative stress markers activity

**Fig. 3:**
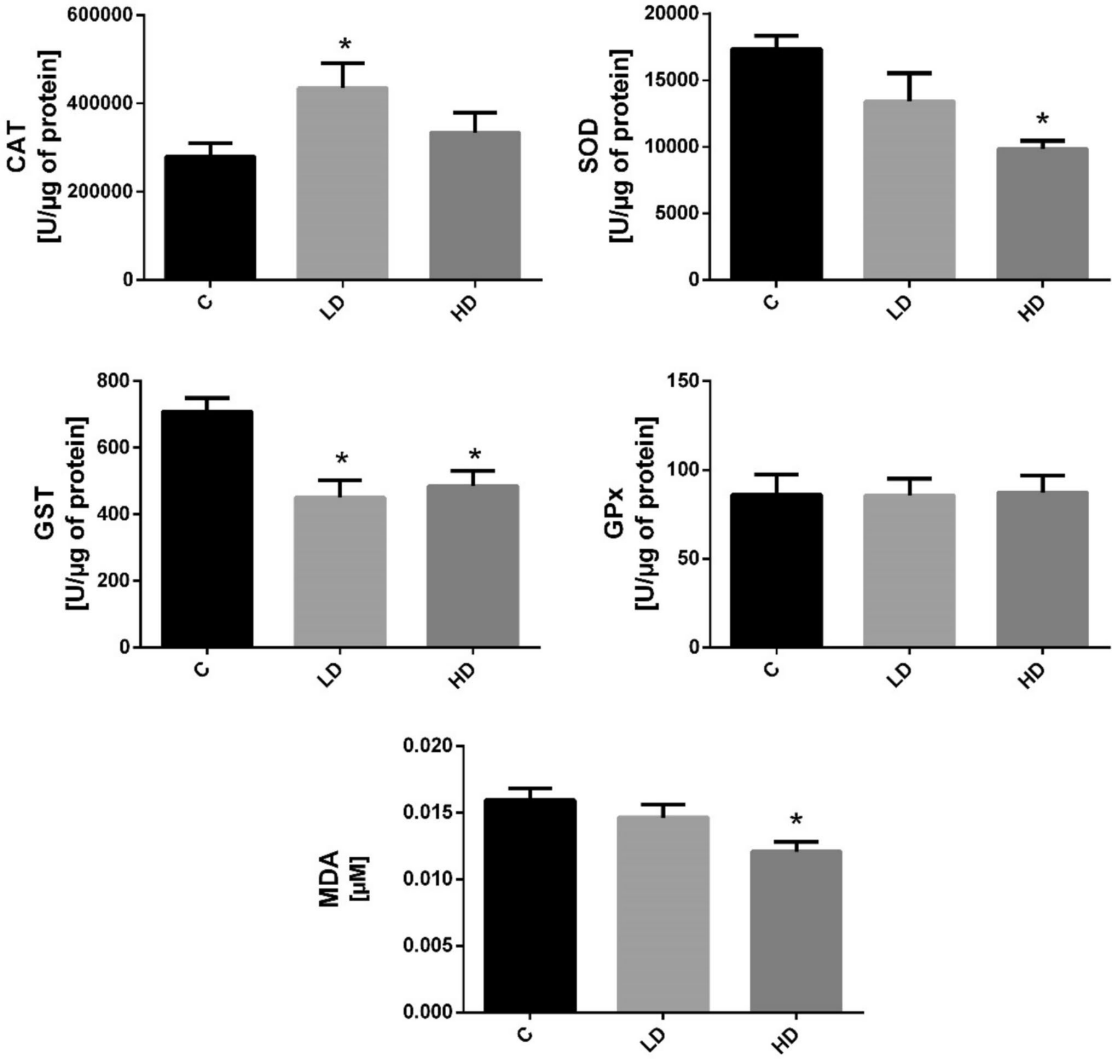
The effect of low (LD) and high (HD) doses of PET microplastics on oxidative stress markers in the liver. The activity levels of catalase (CAT), superoxide dismutase (SOD), glutathione S-transferase (GST), and glutathione peroxidase (GPx) are expressed as U/μg of protein, while malondialdehyde (MDA) concentration is expressed in μM. Data are presented as mean ± SEM. The asterisks indicate statistically significant differences (*p* ≤ 0.05) compared to the control group (C)

We observed a significant increase in catalase (CAT) activity following exposure to a low dose of microplastics (Fig. 3). In contrast, activity of total superoxide dismutase (SOD) decreased, but this was only evident in response to a high dose of microplastics (Fig. 3). Glutathione S-transferase (GST) total activity declined after both low and high doses of microplastics, whereas no significant changes in glutathione peroxidase (GPx) activity were detected following microplastic exposure (Fig. 3). In addition, lipid peroxidation, assessed by malondialdehyde (MDA) levels, was found to be reduced after exposure to a high dose of microplastics (Fig. 3).

### Impact of PET microplastics on the content of selected liver function markers

**Fig. 4:**
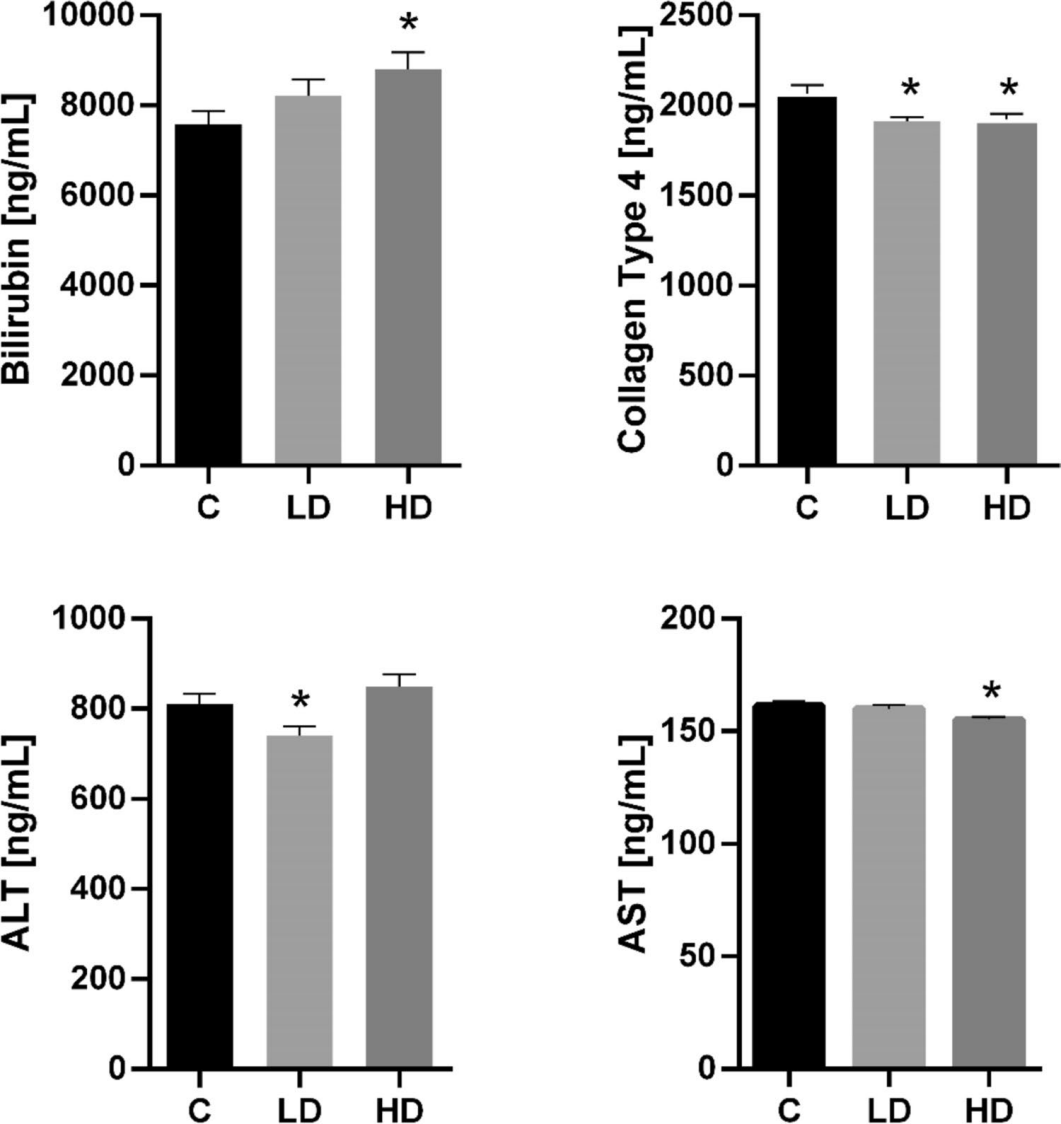
The effect of low (LD) and high (HD) doses of microplastics on liver function and fibrosis-related parameters in tissue homogenates, measured using ELISA. The concentrations of bilirubin, collagen type IV, alanine aminotransferase (ALT), and aspartate aminotransferase (AST) are presented as mean ± SEM. The asterisks indicate statistically significant differences (*p* ≤ 0.05) compared to the control group (C)

We observed increased bilirubin content in liver tissue following exposure to a high dose of microplastics (Fig. 4). In turn, collagen type 4 concentration decreased after both low and high doses of microplastics (Fig. 4). Alanine transaminase (ALT) amount decreased following the low dose, while aspartate aminotransferase (AST) level decreased after the high dose (Fig. 4).

### Real-time PCR

**Fig. 5:**
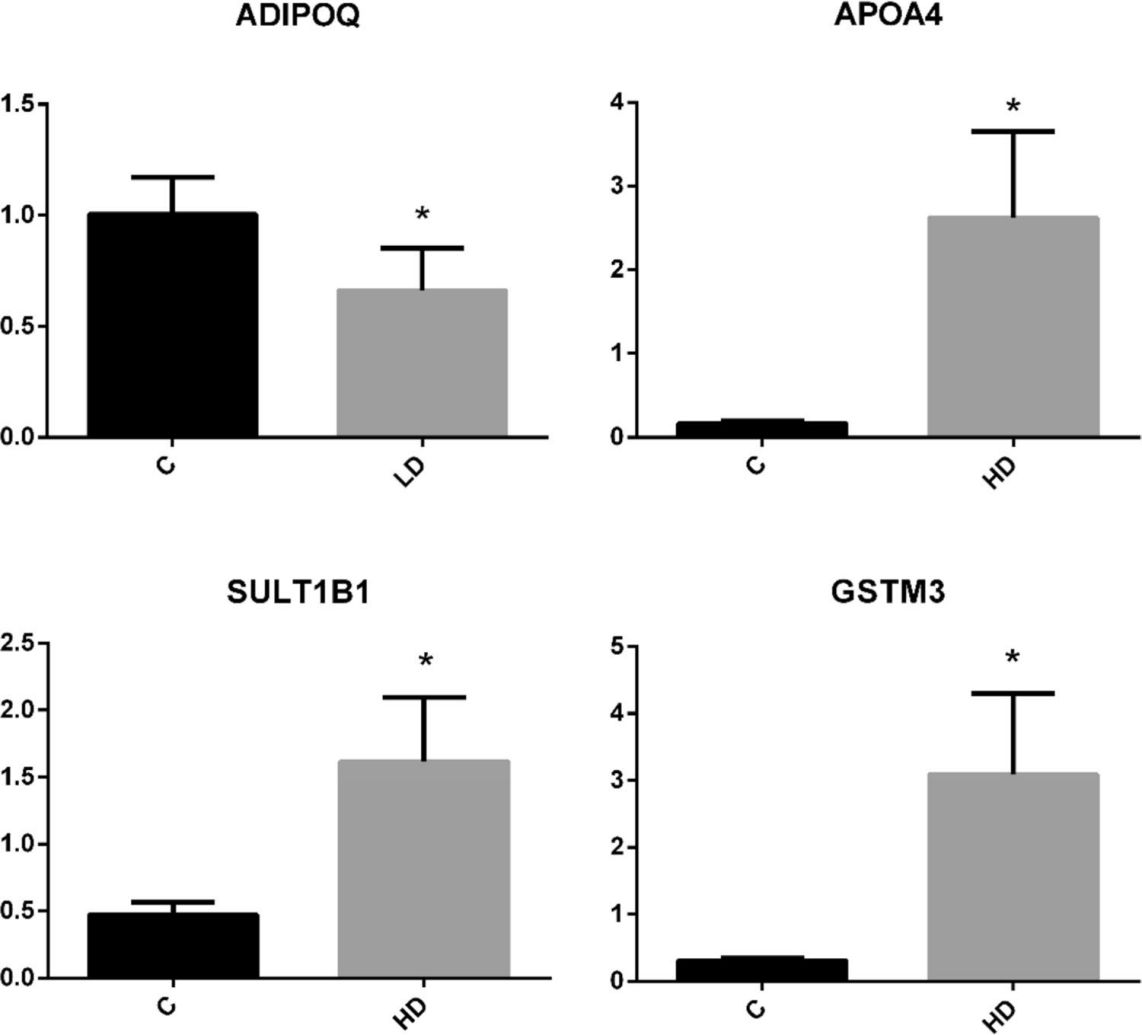
Validation of selected DEGs by real-time PCR. Data are presented as mean ± SEM in arbitrary units. The asterisks indicate statistically significant differences (*p* ≤ 0.05) compared to the control group (C)

The analysis revealed that, in the liver, after exposure to low or high doses of PET MPs, the mRNA abundance of *ADIPOQ, APOA4, SULT1B1,* and *GSTM3* showed expression patterns consistent with the results obtained from the RNA-Seq analysis (Fig. 5).

## Discussion

The liver is a vital organ that plays a crucial role in various physiological processes, including detoxification, metabolic regulation, protein synthesis, bile production, and the storage of glycogen. It is essential for maintaining homeostasis by metabolizing carbohydrates, lipids and proteins and converting harmful substances into less toxic forms, which are then excreted (Kalra et al. 2025). Factors that lead to liver disorders include obesity, insulin resistance, alcohol abuse, viral hepatitis, and genetic predispositions. Recent research suggests that microplastics may play a role in liver damage. When ingested or inhaled, they can accumulate in the liver and induce oxidative stress, inflammation, and alternate lipid metabolism, potentially contributing to the development of fatty liver disease (fat accumulation), fibrosis (collagen deposition), cirrhosis (scar tissue formation), and even liver cancer (Chiang et al. 2024). These pathologies result in significant morbidity and mortality as they disrupt vital metabolic processes, lead to liver failure, and increase the risk of systemic complications.

In the present study, we report for the first time changes in transcriptomic profiles and oxidative stress markers in the liver of pigs exposed to PET microplastics. The results of the transcriptomic analyses indicate that a higher dose of PET exerts a stronger impact on the liver. We observed an upregulation of the expression of several genes involved in cholesterol metabolic processes (*APOA4, HMGCS2, ENSSSCG00000058639*), transferase activity (*ENSSSCG00000053507, SULT1B1, ENSSSCG00000058639, GSTM3, HS3ST2, HMGCS2, ITPKA*) or biological oxidations (*SULT1B1, GSTM3*) after exposure to a high dose of MPs. DEGs detected after a low dose of MPs treatment were involved, for example, in the response to glucose (*ADIPOQ, ABCC8*) or in the cellular lipid metabolic process (*ADIPOQ, PLCE1*). Moreover, we have detected impact of PET MPs on oxidative stress markers activity and content of selected liver function markers.

Available reports indicate that MPs contribute to oxidative stress by inducing the formation of reactive oxygen species (ROS) (Hu and Palić, 2020; Kadac-Czapska et al. 2024). Recent findings have highlighted an additional layer of complexity to MP-induced biological effects, demonstrating that certain members of the human gut microbiota possess enzymatic capabilities enabling the degradation of PET. Recently, the presence of PET hydrolases in the human gut microbiota was demonstrated (Zhang et al. 2024; Zhou et al. 2024). These hydrolases resulted in PET breakdown into mono(2-hydroxyethyl) terephthalic acid (MHET), terephthalic acid (TPA), and ethylene glycol (Zhang et al. 2024; Zhou et al. 2024). It has been shown that TPA promoted the polarization of mouse macrophages toward a pro-inflammatory phenotype and activated the pro-inflammatory NF-κB signaling pathway in mouse adipocytes (Molonia et al. 2022; Zhou et al. 2024). The consequence of immune activation might be elevated ROS production and oxidative stress within the tissue microenvironment. One of the most important antioxidant enzymes involved in the degradation of ROS are SOD, CAT, GST, and GPx (Ighodaro and Akinloye 2018; Matés, 2000). In our study, we observed a decreased activity of SOD and GST after exposure to PET MPs, indicating a reduced ability of cells to neutralize oxidative stress, which can lead to an accumulation of ROS and further oxidative damage (Chaudhary et al. 2023). In contrast, the increased catalase (CAT) activity likely reflects a defense response to oxidative stress induced by PET MPs, aiming to mitigate its effects. It is worth noting that an increase in serum catalase activity was observed in different liver diseases, such as fatty liver or acute liver hepatitis (Góth et al. 1987; Perlemuter et al. 2005). Notably, we found a decrease in malondialdehyde (MDA), an end product of lipid peroxidation that serves as a marker of oxidative stress. Our results suggest that the exposure to PET MPs disrupts the oxidative balance by impairing key antioxidant enzymes (SOD, GST), while CAT is upregulated as a compensatory response. The reduction in MDA levels may indicate alterations in lipid peroxidation dynamics, possibly due to adaptive cellular mechanisms.

The present study revealed that PET MPs change the expression of several genes related to oxidative stress. PET upregulated the expression of oxoglutarate dehydrogenase L (*OGDHL*), which is known to increase ROS production and lipid peroxidation, cumulating during cell apoptosis (Mailloux et al. 2016; Sen et al. 2012). It is worth noting that glutathione S-transferases are important enzymes that protect cells against products of oxidative stress (Mazari et al. 2023; White et al. 2008), and our study demonstrates an increased expression of glutathione S-transferase mu 3 (*GSTM3*) following PET MPs administration, accompanied by a decrease in overall GST enzyme activity. This discrepancy may indicate a compensatory transcriptional response that is insufficient to maintain GST enzymatic function, potentially leading to reduced cellular defense against oxidative stress.

The next gene identified in relation to oxidative stress could be adiponectin, a homeostatic factor responsible for the regulation of glucose levels, lipid metabolism, and insulin sensitivity through its anti-inflammatory, anti-fibrotic, and antioxidant effects. In our study, the exposure to PET MP reduced the expression of adiponectin (*ADIPOQ*) gene. Previous studies have reported that adiponectins are sensitive to oxidative stress and their secretion was diminished in adipocytes under oxidative stress (Begum et al. 2023; Matsuda and Shimomura 2014). High adiponectin levels, both in plasma and under in vitro conditions, inhibit liver fibrosis, reduce the content of pro-inflammatory interleukin-1β (IL-1β), and promote anti-inflammatory interleukin-10 (IL-10), while low levels of adiponectin contribute to hepatic steatosis, inflammation, fibrosis, and metabolic disorders such as insulin resistance and type 2 diabetes (Al-Nbaheen 2022; Handy et al. 2011; Hotta et al. 2001, 2000; Kitamoto et al. 2015; Saxena and Anania 2015; Wolf et al. 2004). Reduced adiponectin plasma levels have been also observed in various liver diseases such as non-alcoholic steatohepatitis (NASH), non-alcoholic fatty liver disease (NAFLD), or hepatocellular carcinoma (HCC) (Gamberi et al. 2018; Shabalala et al. 2020). Our results suggest that PET MPs exposure may reduce adiponectin content in the liver, potentially promoting liver disturbances.

There is increasing evidence that oxidative stress may be an important modulator of liver fibrosis (Li et al. 2015; Ma et al. 2022), and that apolipoprotein A4 (APOA4) and collagen IV could serve as biomarkers in this context (Lønsmann et al. 2023; Wang et al. 2017). Remarkably, increased expression of APOA4 is considered an early marker for liver fibrosis in humans (Wang et al. 2017) and has been observed in various models, including fibrosis in rhesus macaque (Feng et al. 2023) or alcoholic hepatitis in human and mice models (Li et al. 2022). In our study, we also observed a similar increase in *APOA4* expression after exposure to high doses of PET MPs. Moreover, microplastics were detected in liver tissue affected by cirrhosis (Horvatits et al. 2022), supporting the hypothesis that they may play a role in the fibrosis processes. In contrast, collagen type IV—a known marker for fibrosis (Niu and Qi 2022; Stefano et al. 2021; Tsutsumi et al. 1993)—showed a paradoxical decrease in liver tissue levels after exposure to PET MPs in our study. This unexpected decrease could indicate acute liver injury, a phenomenon that has been documented in mouse models during acute liver injury (Hanumegowda et al. 2003), or reflect an imbalance in collagen synthesis and degradation associated with liver cirrhosis (Thiele et al. 2021). Together, these results suggest that microplastics may contribute to the development of liver fibrosis via oxidative stress-mediated mechanisms, while the observed APOA4 upregulation may serve as a potential early biomarker for this pathology. However, the unexpected decrease in collagen IV highlights the complexity of MPs-induced damage, which requires further studies to elucidate their role in both the progression of fibrosis and the onset of acute liver injury.

In the present study, we reported a significant increase in bilirubin levels in liver tissue after exposure to a high dose of PET MPs. Bilirubin, a product of heme catabolism, is widely recognized as a marker for liver disease (Guerra Ruiz et al. 2021). Elevated serum levels may result from increased uptake by the liver or impaired excretion into the bile (Guerra Ruiz et al. 2021). Remarkably, bilirubin also possesses antioxidant properties and protects against lipid peroxidation (Hansen et al. 2020; Tomaro et al. 2002; Weaver et al. 2018; Zelenka et al. 2012). The elevated bilirubin levels in liver tissue in our study may represent a compensatory response to the oxidative stress induced by PET MPs. In addition to the change in bilirubin levels, we noted a decrease in alanine transaminase (ALT; after low doses of PET MPs) and aspartate aminotransferase (AST; after high doses of PET MPs) levels in liver tissue. Both ALT and AST are mainly located in the cytosol of hepatocytes (Moriles et al. 2025) and are responsible for amino acid metabolism (McGill 2016). The decrease in tissue ALT and AST levels may suggest hepatocytes degradation and the release of these transaminases from liver tissue into the general circulation (Thakur et al. 2024). It should also be considered that diseases, like cholestasis, may lead to changes in liver metabolism and abnormal synthesis of enzymes (Giannini et al. 2005). Our findings suggest that changes in tissue aminotransferases levels without corresponding changes in serum biochemistry (Mierzejewski et al. 2025) may indicate metabolic disturbances in the liver, perhaps related to gluconeogenesis. Interestingly, accelerated fatty acid oxidation increases the synthesis of enzymes related to gluconeogenesis, including AST and ALT (Kobayashi et al. 2020). However, our study showed a decrease in malondialdehyde (MDA), an end product of lipid peroxidation, accompanied by reduced activity of these transaminases. These observations may suggest impaired liver metabolism, particularly lipid metabolism and gluconeogenesis.

Recent studies suggest a link between exposure to microplastics and carcinogenesis (Goswami et al. 2024). In the present study, we found decreased expression of the gene phospholipase C epsilon 1 (*PLCE1*) after PET MPs. This reduction aligns with findings in hepatocellular carcinoma tissue and cell lines, where the expression and protein levels of PLCE1 were significantly lower in cancer (Cheng et al. 2016). Research indicates that lower expression of *PLCE1* increases the risk of colorectal cancer (Wang et al. 2014), and overexpression of *PLCE*1 significantly reduces the proliferation of colorectal cancer cells and decreases their degree of malignancy (Wang et al. 2012). In turn, loss of *PLCE1* reduced the invasion and proliferation ability of esophageal cancer cells in vitro (Zhai et al. 2017) and promoted apoptosis in human tongue squamous cell carcinoma (Abulaiti et al. 2022). These findings suggest that the role of PLCE1 is tissue-specific, and its downregulation after MP treatment may reveal novel mechanisms of MP-induced toxicity related to carcinogenesis.

To summarize, our study demonstrates possible mechanisms by which PET microplastics trigger liver physiology. Exposure to microplastics has effects on important biological signaling pathways, including oxidative stress and metabolic processes. These findings suggest that ingestion of PET microplastics may pose a significant health risk to humans by disrupting liver function and metabolic homeostasis. Long-term exposure may contribute to the development of chronic liver disease and other systemic disorders, highlighting the importance of minimizing microplastic contamination in food. Therefore, strict monitoring of microplastic contamination, especially in edible products, is necessary. Future research should prioritize mechanistic studies that allow a comprehensive analysis of microplastic-induced changes in liver function.

## Supplementary Information

Below is the link to the electronic supplementary material.

Supplementary file1 (XLSX 6868 KB)

Supplementary file2 (XLSX 45 KB)

## Data Availability

The raw data generated for this study can be found in the European Nucleotide Archive (ENA) with the accession number PRJEB90094.

## References

[CR1] Abarghouei S, Hedayati A, Raeisi M, Hadavand BS, Rezaei H, Abed-Elmdoust A (2021) Size-dependent effects of microplastic on uptake, immune system, related gene expression and histopathology of goldfish (Carassius auratus). Chemosphere 276:129977. 10.1016/j.chemosphere.2021.129977 33684862 10.1016/j.chemosphere.2021.129977

[CR2] Abulaiti Z, Chen L, Xiao Q, Aimaier A, Ma Y, He S, Zhang J, Xu J, Cui X (2022) PLCE1 as a diagnostic and prognostic biomarker by promoting the growth and progression of oral squamous cell carcinoma. J Oral Pathol Med 51(9):771–779. 10.1111/jop.13349 36065133 10.1111/jop.13349

[CR3] Al-Nbaheen MS (2022) Effect of genetic variations in the ADIPOQ gene on susceptibility to type 2 diabetes mellitus. Diabetes Metab Syndr Obes 15:2753–2761. 10.2147/DMSO.S377057 36101664 10.2147/DMSO.S377057 PMC9464438

[CR4] Begum M, Choubey M, Tirumalasetty MB, Arbee S, Mohib MM, Wahiduzzaman M, Mamun MA, Uddin MB, Mohiuddin MS (2023) Adiponectin: a promising target for the treatment of diabetes and its complications. Life (Basel, Switzerland) 13(11):2213. 10.3390/life13112213 38004353 10.3390/life13112213 PMC10672343

[CR5] Chaudhary P, Janmeda P, Docea AO, Yeskaliyeva B, Abdull Razis AF, Modu B, Calina D, Sharifi-Rad J (2023) Oxidative stress, free radicals and antioxidants: potential crosstalk in the pathophysiology of human diseases. Front Chem 11:1158198. 10.3389/fchem.2023.1158198 37234200 10.3389/fchem.2023.1158198 PMC10206224

[CR6] Chen X, Zhuang J, Chen Q, Xu L, Yue X, Qiao D (2022) Chronic exposure to polyvinyl chloride microplastics induces liver injury and gut microbiota dysbiosis based on the integration of liver transcriptome profiles and full-length 16S rRNA sequencing data. Sci Total Environ 839:155984. 10.1016/j.scitotenv.2022.155984 35588832 10.1016/j.scitotenv.2022.155984

[CR7] Cheng Y, Xing S-G, Jia W-D, Huang M, Bian N-N (2016) Low *PLCE1* levels are correlated with poor prognosis in hepatocellular carcinoma. OncoTargets Ther 10:47–54. 10.2147/OTT.S126401 10.2147/OTT.S126401 PMC5182043 28031722

[CR8] Chiang CC, Yeh H, Shiu RF, Chin WC, Yen TH (2024) Impact of microplastics and nanoplastics on liver health: Current understanding and future research directions. World J Gastroenterol 30(9):1011–1017. 10.3748/wjg.v30.i9.1011 38577182 10.3748/wjg.v30.i9.1011 PMC10989496

[CR9] Choi JS, Jung Y-J, Hong N-H, Hong SH, Park J-W (2018) Toxicological effects of irregularly shaped and spherical microplastics in a marine teleost, the sheepshead minnow (*Cyprinodon variegatus*). Mar Pollut Bull 129(1):231–240. 10.1016/j.marpolbul.2018.02.039 29680542 10.1016/j.marpolbul.2018.02.039

[CR10] Deng Y, Zhang Y, Lemos B, Ren H (2017) Tissue accumulation of microplastics in mice and biomarker responses suggest widespread health risks of exposure. Sci Rep 7:46687. 10.1038/srep46687 28436478 10.1038/srep46687 PMC5402289

[CR11] Djouina M, Vignal C, Dehaut A, Caboche S, Hirt N, Waxin C, Himber C, Beury D, Hot D, Dubuquoy L, Launay D, Duflos G, Body-Malapel M (2022) Oral exposure to polyethylene microplastics alters gut morphology, immune response, and microbiota composition in mice. Environ Res 212(Pt B):113230. 10.1016/j.envres.2022.113230 35398082 10.1016/j.envres.2022.113230

[CR12] Feng T, Lai C, Yuan Q, Yang W, Yao Y, Du M, Zhong D, Wang S, Yang Q, Shang J, Shi Y, Huang X (2023) Non-invasive assessment of liver fibrosis by serum metabolites in non-human primates and human patients. iScience 26(9):107538. 10.1016/j.isci.2023.107538 37636059 10.1016/j.isci.2023.107538 PMC10448158

[CR13] Fournier E, Etienne-Mesmin L, Grootaert C, Jelsbak L, Syberg K, Blanquet-Diot S, Mercier-Bonin M (2021) Microplastics in the human digestive environment: A focus on the potential and challenges facing in vitro gut model development. J Hazard Mater 415:125632. 10.1016/j.jhazmat.2021.125632 33770682 10.1016/j.jhazmat.2021.125632

[CR14] Gałęcka I, Szyryńska N, Całka J (2024) Influence of polyethylene terephthalate (PET) microplastic on selected active substances in the intramural neurons of the porcine duodenum. Part Fibre Toxicol 21(1):5. 10.1186/s12989-024-00566-w 38321545 10.1186/s12989-024-00566-w PMC10845528

[CR15] Gałęcka I, Rychlik A, Całka J (2025) Influence of selected dosages of plastic microparticles on the porcine fecal microbiome. Sci Rep 15(1):1269. 10.1038/s41598-024-80337-x 39779716 10.1038/s41598-024-80337-x PMC11711237

[CR16] Gamberi T, Magherini F, Modesti A, Fiaschi T (2018) Adiponectin signaling pathways in liver diseases. Biomedicines 6(2):52. 10.3390/biomedicines6020052 29735928 10.3390/biomedicines6020052 PMC6027295

[CR17] Giannini EG, Testa R, Savarino V (2005) Liver enzyme alteration: a guide for clinicians. CMAJ: Can Med Assoc J = J de l’Association medicale canadienne 172(3):367–379. 10.1503/cmaj.1040752 10.1503/cmaj.1040752 PMC545762 15684121

[CR18] Goswami S, Adhikary S, Bhattacharya S, Agarwal R, Ganguly A, Nanda S, Rajak P (2024) The alarming link between environmental microplastic s and health hazards with special emphasis on cancer. Life Sci 355:122937. 10.1016/j.lfs.2024.122937 39103046 10.1016/j.lfs.2024.122937

[CR19] Góth L, Mészáros I, Németh H (1987) Serum catalase enzyme activity in liver diseases. Acta Biol Hung 38(2):287–290 3454088

[CR20] Guerra Ruiz AR, Crespo J, López Martínez RM, Iruzubieta P, Casals Mercadal G, Lalana Garcés M, Lavin B, Morales Ruiz M (2021) Measurement and clinical usefulness of bilirubin in liver disease. Adv Lab Med 2(3):352–372. 10.1515/almed-2021-0047 37362415 10.1515/almed-2021-0047 PMC10197288

[CR21] Handy JA, Fu PP, Kumar P, Mells JE, Sharma S, Saxena NK, Anania FA (2011) Adiponectin inhibits leptin signalling via multiple mechanisms to exert protective effects against hepatic fibrosis. Biochem J 440:385–395. 10.1042/BJ20102148 21846328 10.1042/BJ20102148 PMC3226855

[CR22] Hansen TWR, Wong RJ, Stevenson DK (2020) Molecular physiology and pathophysiology of bilirubin handling by the blood, Liver, intestine, and brain in the newborn. Physiol Rev 100(3):1291–1346. 10.1152/physrev.00004.2019 32401177 10.1152/physrev.00004.2019

[CR23] Hanumegowda UM, Copple BL, Shibuya M, Malle E, Ganey PE, Roth RA (2003) Basement membrane and matrix metalloproteinases in monocrotaline-induced liver injury. Toxicol Sci 76:237–246. 10.1093/toxsci/kfg222 12970574 10.1093/toxsci/kfg222

[CR24] Horvatits T, Tamminga M, Liu B, Sebode M, Carambia A, Fischer L, Püschel K, Huber S, Fischer EK (2022) Microplastics detected in cirrhotic liver tissue. EBioMedicine 82:104147 35835713 10.1016/j.ebiom.2022.104147 PMC9386716

[CR25] Hotta K, Funahashi T, Arita Y, Takahashi M, Matsuda M, Okamoto Y, Iwahashi H, Kuriyama H, Ouchi N, Maeda K, Nishida M, Kihara S, Sakai N, Nakajima T, Hasegawa K, Muraguchi M, Ohmoto Y, Nakamura T, Yamashita S, Hanafusa T, Matsuzawa Y (2000) Plasma concentrations of a novel, adipose-specific protein, adiponectin, in type 2 diabetic patients. Arterioscler Thromb Vasc Biol 20(6):1595–1599 10845877 10.1161/01.atv.20.6.1595

[CR26] Hotta K, Funahashi T, Bodkin NL, Ortmeyer HK, Arita Y, Hansen BC, Matsuzawa Y (2001) Circulating concentrations of the adipocyte protein adiponectin are decreased in parallel with reduced insulin sensitivity during the progression to type 2 diabetes in rhesus monkeys. Diabetes 50:1126–1133. 10.2337/diabetes.50.5.1126 11334417 10.2337/diabetes.50.5.1126

[CR27] Hu M, Palić D (2020) Micro- and nano-plastics activation of oxidative and inflammatory adverse outcome pathways. Redox Biol 37:101620. 10.1016/j.redox.2020.101620 32863185 10.1016/j.redox.2020.101620 PMC7767742

[CR28] Ighodaro OM, Akinloye OA (2018) First line defence antioxidants-superoxide dismutase (SOD), catalase (CAT) and glutathione peroxidase (GPX): their fundamental role in the entire antioxidant defence grid. Alexandria J Med 54:287–293. 10.1016/j.ajme.2017.09.001

[CR29] Kadac-Czapska K, Ośko J, Knez E, Grembecka M (2024) Microplastics and oxidative stress—current problems and prospects. Antioxidants 13(5):579. 10.3390/antiox13050579 38790684 10.3390/antiox13050579 PMC11117644

[CR30] Kalra A, Yetiskul E, Wehrle CJ, Tuma F (2025) Physiology, Liver. Stat Pearls Publishing, Treasure Island, FL 30571059

[CR31] Kedzierski M, Lechat B, Sire O, Le Maguer G, Le Tilly V, Bruzaud S (2020) Microplastic contamination of packaged meat: Occurrence and associated risks. Food Packag Shelf Life 24:100489. 10.1016/j.fpsl.2020.100489

[CR32] Kitamoto A, Kitamoto T, So R, Matsuo T, Nakata Y, Hyogo H, Ochi H, Nakamura T, Kamohara S, Miyatake N, Kotani K, Mineo I, Wada J, Ogawa Y, Yoneda M, Nakajima A, Funahashi T, Miyazaki S, Tokunaga K, Masuzaki H, Ueno T, Chayama K, Hamaguchi K, Yamada K, Hanafusa T, Oikawa S, Sakata T, Tanaka K, Matsuzawa Y, Hotta K (2015) ADIPOQ polymorphisms are associated with insulin resistance in Japanese women. Endocr J 62(6):513–521. 10.1507/endocrj.EJ14-0574 25832963 10.1507/endocrj.EJ14-0574

[CR33] Kobayashi A, Suzuki Y, Sugai S (2020) Specificity of transaminase activities in the prediction of drug-induced hepatotoxicity. J Toxicol Sci 45(9):515–537. 10.2131/jts.45.515 32879252 10.2131/jts.45.515

[CR34] Kurchaba N, Cassone BJ, Northam C, Ardelli BF, Lemoine CMR (2020) Effects of mp polyethylene microparticles on microbiome and inflammatory response of larval zebrafish. Toxics 8(3):55. 10.3390/toxics8030055 32796641 10.3390/toxics8030055 PMC7560425

[CR35] Leslie HA, van Velzen MJM, Brandsma SH, Vethaak AD, Garcia-Vallejo JJ, Lamoree MH (2022) Discovery and quantification of plastic particle pollution in human blood. Environ Int 163:107199. 10.1016/j.envint.2022.107199 35367073 10.1016/j.envint.2022.107199

[CR36] Li S, Tan H-Y, Wang N, Zhang Z-J, Lao L, Wong C-W, Feng Y (2015) The role of oxidative stress and antioxidants in liver diseases. Int J Mol Sci 16(11):26087–26124. 10.3390/ijms161125942 26540040 10.3390/ijms161125942 PMC4661801

[CR37] Li W-H, Zhang L, Li Y-Y, Wang X-Y, Li J-L, Zhao S-N, Ni M-Q, Li Q, Sun H (2022) Apolipoprotein A-IV has bi-functional actions in alcoholic hepatitis by regulating hepatocyte injury and immune cell infiltration. Int J Mol Sci 24(1):670. 10.3390/ijms24010670 36614113 10.3390/ijms24010670 PMC9820766

[CR38] Limonta G, Mancia A, Benkhalqui A, Bertolucci C, Abelli L, Cristina Fossi M, Panti C (2019) Microplastics induce transcriptional changes, immune response and behavioral alterations in adult zebrafish. Sci Rep 9(1):15775. 10.1038/s41598-019-52292-5 31673028 10.1038/s41598-019-52292-5 PMC6823372

[CR39] Liu C, Chen S, Chu J, Yang Y, Yuan B, Zhang H (2024) Multi-omics analysis reveals the toxicity of polyvinyl chloride microplastics toward BEAS-2B cells. Toxics 12(6):399. 10.3390/toxics12060399 38922079 10.3390/toxics12060399 PMC11209221

[CR40] Lønsmann I, Grove JI, Haider A, Kaye P, Karsdal MA, Leeming DJ, Aithal GP (2023) Biomarkers of type IV collagen turnover reflect disease activity in patients with early-stage non-alcoholic fatty liver (NAFL). Biology 12(8):1087. 10.3390/biology12081087 37626973 10.3390/biology12081087 PMC10451710

[CR41] Lu L, Luo T, Zhao Y, Cai C, Fu Z, Jin Y (2019) Interaction between microplastics and microorganism as well as gut microbiota: a consideration on environmental animal and human health. Sci Total Environ 667:94–100. 10.1016/J.SCITOTENV.2019.02.380 30826685 10.1016/j.scitotenv.2019.02.380

[CR42] Lunney JK, Van Goor A, Walker KE, Hailstock T, Franklin J, Dai C (2021) Importance of the pig as a human biomedical model. Sci Transl Med 13(621):eabd5758. 10.1126/scitranslmed.abd5758 34818055 10.1126/scitranslmed.abd5758

[CR43] Luo T, Weng Y, Huang Z, Zhao Y, Jin Y (2021) Combined hepatotoxicity of imidacloprid and microplastics in adult zebrafish: Endpoints at gene transcription. Comp Biochem Physiol Toxicol Pharmacol CBP 246:109043. 10.1016/j.cbpc.2021.109043 10.1016/j.cbpc.2021.109043 33862234

[CR44] Ma Y, Lee G, Heo SY, Roh YS (2022) Oxidative stress is a key modulator in the development of nonalcoholic fatty liver disease. Antioxidants 11(1):91. 10.3390/antiox11010091 10.3390/antiox11010091 PMC8772974 35052595

[CR45] Mailloux RJ, Craig Ayre D, Christian SL (2016) Induction of mitochondrial reactive oxygen species production by GSH mediated s-glutathionylation of 2-oxoglutarate dehydrogenase. Redox Biol 8:285–297. 10.1016/j.redox.2016.02.002 26928132 10.1016/j.redox.2016.02.002 PMC4776629

[CR46] Marcellus KA, Bugiel S, Nunnikhoven A, Curran I, Gill SS (2024) Polystyrene nano- and microplastic particles induce an inflammatory gene expression profile in rat neural stem cell-derived astrocytes in vitro. Nanomaterials 14(5):429. 10.3390/nano14050429 38470760 10.3390/nano14050429 PMC10935329

[CR47] Matés JM (2000) Effects of antioxidant enzymes in the molecular control of reactive oxygen species toxicology. Toxicology 153(1–3):83–104. 10.1016/s0300-483x(00)00306-1 11090949 10.1016/s0300-483x(00)00306-1

[CR48] Matsuda M, Shimomura I (2014) Roles of adiponectin and oxidative stress in obesity-associated metabolic and cardiovascular diseases. Rev Endocr Metab Disord 15(1):1–10. 10.1007/s11154-013-9271-7 24026768 10.1007/s11154-013-9271-7

[CR49] Mazari AMA, Zhang L, Ye Z-W, Zhang J, Tew KD, Townsend DM (2023) The multifaceted role of glutathione S-transferases in health and disease. Biomolecules 13(4):688. 10.3390/biom13040688 37189435 10.3390/biom13040688 PMC10136111

[CR50] McGill MR (2016) The past and present of serum aminotransferases and the future of liver injury biomarkers. EXCLI J 15:817–828. 10.17179/excli2016-800 28337112 10.17179/excli2016-800 PMC5318690

[CR51] Mierzejewski K, Paukszto Ł, Kurzyńska A, Kunicka Z, Jastrzȩbski JP, Bogacka I (2021) Transcriptome analysis of porcine endometrium after LPS-induced inflammation: effects of the PPAR-gamma ligands in vitro. Biol Reprod 104(1):130–143. 10.1093/biolre/ioaa200 33112378 10.1093/biolre/ioaa200

[CR52] Mierzejewski K, Kurzyńska A, Golubska M, Całka J, Gałęcka I, Szabelski M, Paukszto L, Andronowska A, Bogacka I (2023) New insights into the potential effects of PET microplastics on organisms via extracellular vesicle-mediated communication. Sci Total Environ 904:166967. 10.1016/j.scitotenv.2023.166967 37699490 10.1016/j.scitotenv.2023.166967

[CR53] Mierzejewski K, Kurzyńska A, Golubska M, Gałęcka I, Całka J, Bogacka I (2025) Oral exposure to PET microplastics induces the pancreatic immune response and oxidative stress in immature pigs. BMC Genomics 26(1):578. 10.1186/s12864-025-11760-1 40597598 10.1186/s12864-025-11760-1 PMC12211908

[CR54] Molonia MS, Muscarà C, Speciale A, Salamone FL, Toscano G, Saija A, Cimino F (2022) The p-phthalates terephthalic acid and dimethyl terephthalate used in the manufacture of PET induce *in vitro* adipocytes dysfunction by altering adipogenesis and thermogenesis mechanisms. Molecules 27(21):7645. 10.3390/molecules27217645 36364480 10.3390/molecules27217645 PMC9656719

[CR55] Moriles KE, Zubair M, Azer SA (2025) Alanine Aminotransferase (ALT) Test. Stat Pearls Publishing, Treasure Island, FL 32644704

[CR56] Nisticò R (2020) Polyethylene terephthalate (PET) in the packaging industry. Polym Test. 10.1016/j.polymertesting.2020.106707

[CR57] Niu A, Qi T (2022) Diagnostic significance of serum type IV collagen (IVC) combined with aspartate aminotransferase (AST)/alanine aminotransferase (ALT) ratio in liver fibrosis. Ann Transl Med 10(24):1310. 10.21037/atm-22-5010 36660657 10.21037/atm-22-5010 PMC9843355

[CR58] Oliveri Conti G, Ferrante M, Banni M, Favara C, Nicolosi I, Cristaldi A, Fiore M, Zuccarello P (2020) Micro- and nano-plastics in edible fruit and vegetables. The first diet risks assessment for the general population. Environ Res 187:109677. 10.1016/j.envres.2020.109677 32454310 10.1016/j.envres.2020.109677

[CR59] Patra I, Huy DTN, Alsaikhan F, Opulencia MJC, Van Tuan P, Nurmatova KC, Majdi A, Shoukat S, Yasin G, Margiana R, Walker TR, Karbalaei S (2022) Toxic effects on enzymatic activity, gene expression and histopathological biomarkers in organisms exposed to microplastics and nanoplastics: a review. Environ Sci Eur 34:80. 10.1186/s12302-022-00652-w

[CR60] Perlemuter G, Davit-Spraul A, Cosson C, Conti M, Bigorgne A, Paradis V, Corre MP, Prat L, Kuoch V, Basdevant A, Pelletier G, Oppert JM, Buffet C (2005) Increase in liver antioxidant enzyme activities in non-alcoholic fatty liver disease. Liver Int 25(5):946–953. 10.1111/j.1478-3231.2005.01126.x 16162151 10.1111/j.1478-3231.2005.01126.x

[CR61] PlasticsEurope, 2020. Plastics: the facts 2020: An Analysis of European Plastics Production, Demand and Waste Data. https://plasticseurope.org/knowledge-hub/plastics-the-facts-2020/. Accessed on 21 Mar 2025

[CR62] Prata JC, da Costa JP, Lopes I, Duarte AC, Rocha-Santos T (2020) Environmental exposure to microplastics: an overview on possible human health effects. Sci Total Environ 702:134455. 10.1016/j.scitotenv.2019.134455 31733547 10.1016/j.scitotenv.2019.134455

[CR63] Saxena NK, Anania FA (2015) Adipocytokines and hepatic fibrosis. Trends Endocrinol Metab 26(3):153–161. 10.1016/j.tem.2015.01.002 25656826 10.1016/j.tem.2015.01.002 PMC4718075

[CR64] Schwabl P, Koppel S, Konigshofer P, Bucsics T, Trauner M, Reiberger T, Liebmann B (2019) Detection of various microplastics in human stool: a prospective case series. Ann Intern Med 171(7):453–457. 10.7326/M19-0618 31476765 10.7326/M19-0618

[CR65] Sen T, Sen N, Noordhuis MG, Ravi R, Wu TC, Ha PK, Sidransky D, Hoque MO (2012) OGDHL is a modifier of AKT-dependent signaling and NF-κB function. PLoS ONE 7(11):e48770. 10.1371/journal.pone.0048770 23152800 10.1371/journal.pone.0048770 PMC3495966

[CR66] Senathirajah K, Attwood S, Bhagwat G, Carbery M, Wilson S, Palanisami T (2021) Estimation of the mass of microplastics ingested – a pivotal first step towards human health risk assessment. J Hazard Mater 404(Pt B):124004. 10.1016/j.jhazmat.2020.124004 33130380 10.1016/j.jhazmat.2020.124004

[CR67] Shabalala SC, Dludla PV, Mabasa L, Kappo AP, Basson AK, Pheiffer C, Johnson R (2020) The effect of adiponectin in the pathogenesis of non-alcoholic fatty liver disease (NAFLD) and the potential role of polyphenols in the modulation of adiponectin signaling. Biomed Pharmacother 131:110785. 10.1016/j.biopha.2020.110785 33152943 10.1016/j.biopha.2020.110785

[CR68] Stefano JT, Guedes LV, de Souza AAA, Vanni DS, Alves VAF, Carrilho FJ, Largura A, Arrese M, Oliveira CP (2021) Usefulness of collagen type IV in the detection of significant liver fibrosis in nonalcoholic fatty liver disease. Ann Hepatol 20:100253. 10.1016/j.aohep.2020.08.070 32949785 10.1016/j.aohep.2020.08.070

[CR69] Sun H, Chen N, Yang X, Xia Y, Wu D (2021) Effects induced by polyethylene microplastics oral exposure on colon mucin release, inflammation, gut microflora composition and metabolism in mice. Ecotoxicol Environ Saf 220:112340. 10.1016/J.ECOENV.2021.112340 34015635 10.1016/j.ecoenv.2021.112340

[CR70] Tang KHD (2025) Genotoxicity of microplastics on living organisms: effects on chromosomes DNA and gene expression. Environments 12(1):10. 10.3390/environments12010010

[CR71] Thakur S, Kumar V, Das R, Sharma V, Mehta DK (2024) Biomarkers of hepatic toxicity: an overview. Curr Ther Res Clin Exp 100:100737. 10.1016/j.curtheres.2024.100737 38860148 10.1016/j.curtheres.2024.100737 PMC11163176

[CR72] Thiele M, Johansen S, Gudmann NS, Madsen B, Kjærgaard M, Nielsen MJ, Leeming DJ, Jacobsen S, Bendtsen F, Møller S, Detlefsen S, Karsdal M, Krag A, Arumugam M, Bork P, Hansen T, Anastasiadou E, Hartoft C, Israelsen H, Legido-Quigley C, Melberg HO, Trebicka J (2021) Progressive alcohol-related liver fibrosis is characterised by imbalanced collagen formation and degradation. Aliment Pharmacol Ther 54(8):1070–1080. 10.1111/apt.16567 34428307 10.1111/apt.16567 PMC9292476

[CR73] Tomaro ML, Del AM, Batlle C (2002) Bilirubin: its role in cytoprotection against oxidative stress. Int J Biochem Cell Biol 34(3):216–220 11849989 10.1016/s1357-2725(01)00130-3

[CR74] Toussaint B, Raffael B, Angers-Loustau A, Gilliland D, Kestens V, Petrillo M, Rio-Echevarria IM, Van den Eede G (2019) Review of micro- and nanoplastic contamination in the food chain. Food Addit Contam Part A Chem Anal Control Expo Risk Assess 36(5):639–673. 10.1080/19440049.2019.1583381 30985273 10.1080/19440049.2019.1583381

[CR75] Tsutsumi M, Urashima S, Mtsuda Y, Takase S (1993) Changes in type IV collagen content in livers of patients with alcoholic liver disease. Hepatology 17(5):820–827 8491450

[CR76] Wang X, Zhou C, Qiu G, Yang Y, Yan D, Xing T, Fan J, Tang H, Peng Z (2012) Phospholipase C epsilon plays a suppressive role in incidence of colorectal cancer. Med Oncol 29:1051–1058. 10.1007/s12032-011-9981-1 21667163 10.1007/s12032-011-9981-1

[CR77] Wang Q, Chen P, Chen D, Liu F, Pan W (2014) Association between phospholipase C epsilon gene (PLCE1) polymorphism and colorectal cancer risk in a Chinese population. J Int Med Res 42:270–281. 10.1177/0300060513492484 24496148 10.1177/0300060513492484

[CR78] Wang P-W, Hung Y-C, Wu T-H, Chen M-H, Yeh C-T, Pan T-L (2017) Proteome-based identification of apolipoprotein A-IV as an early diagnostic biomarker in liver fibrosis. Oncotarget 8(51):88951–88964. 10.18632/oncotarget.21627 29179490 10.18632/oncotarget.21627 PMC5687660

[CR79] Weaver L, Hamoud A-R, David X, Stec E, Hinds TD (2018) Biliverdin reductase and bilirubin in hepatic disease. Am J Physiol Gastrointest Liver Physiol 314(6):G668–G676. 10.1152/ajpgi.00026.2018 29494209 10.1152/ajpgi.00026.2018 PMC6032063

[CR80] White DL, Li D, Nurgalieva Z, El-Serag HB (2008) Genetic variants of glutathione S-transferase as possible risk factors for hepatocellular carcinoma: a HuGE systematic review and meta-analysis. Am J Epidemiol 167(4):377–389. 10.1093/aje/kwm315 18065725 10.1093/aje/kwm315

[CR81] Wolf AM, Wolf D, Rumpold H, Enrich B, Tilg H (2004) Adiponectin induces the anti-inflammatory cytokines IL-10 and IL-1RA in human leukocytes. Biochem Biophys Res Commun 323(2):630–635. 10.1016/j.bbrc.2004.08.145 15369797 10.1016/j.bbrc.2004.08.145

[CR82] Xu K, Zhang Y, Huang Y, Wang J (2021) Toxicological effects of microplastics and phenanthrene to zebrafish (*Danio rerio*). Sci Total Environ 757:143730. 10.1016/j.scitotenv.2020.143730 33277007 10.1016/j.scitotenv.2020.143730

[CR83] Yan Z, Liu Y, Zhang T, Zhang F, Ren H, Zhang Y (2022) Analysis of microplastics in human feces reveals a correlation between fecal microplastics and inflammatory bowel disease status. Environ Sci Technol 56(1):414–421. 10.1021/acs.est.1c03924 34935363 10.1021/acs.est.1c03924

[CR84] Yang YF, Chen CY, Lu TH, Liao CM (2019) Toxicity-based toxicokinetic/toxicodynamic assessment for bioaccumulation of polystyrene microplastics in mice. J Hazard Mater 366:703–713. 10.1016/j.jhazmat.2018.12.048 30583240 10.1016/j.jhazmat.2018.12.048

[CR85] Ye G, Zhang X, Liu X, Liao X, Zhang H, Yan C, Lin Y, Huang Q (2021) Polystyrene microplastics induce metabolic disturbances in marine medaka (*Oryzias melastigmas*) liver. Sci Total Environ 782:146885. 10.1016/j.scitotenv.2021.146885

[CR86] Yee MSL, Hii LW, Looi CK, Lim WM, Wong SF, Kok YY, Tan BK, Wong CY, Leong CO (2021) Impact of microplastics and nanoplastics on human health. Nanomaterials 11:1–23. 10.3390/nano11020496 10.3390/nano11020496 PMC7920297 33669327

[CR87] Zelenka J, Muchova L, Zelenkova M, Vanova K, Vreman HJ, Wong RJ, Vitek L (2012) Intracellular accumulation of bilirubin as a defense mechanism against increased oxidative stress. Biochimie 94:1821–1827. 10.1016/j.biochi.2012.04.026 22580386 10.1016/j.biochi.2012.04.026

[CR88] Zhai S, Liu C, Zhang L, Zhu J, Guo J, Zhang J, Chen Z, Zhou W, Chang T, Xu S, Qi Y, Zhuang T, Yu N, Wang W, Wang H, Yu S, Li X (2017) PLCE1 promotes esophageal cancer cell progression by maintaining the transcriptional activity of Snail. Neoplasia 19:154–164. 10.1016/j.neo.2016.12.007 28147304 10.1016/j.neo.2016.12.007 PMC5279705

[CR89] Zhang J, Wang L, Trasande L, Kannan K (2021) Occurrence of polyethylene terephthalate and polycarbonate microplastics in infant and adult feces. Environ Sci Technol Lett 8:989–994. 10.1021/acs.estlett.1c00559 10.1021/acs.est.9b03912 31525038

[CR90] Zhang G, Du J, Zhang C, Zhao Z, Chen Y, Liu M, Chen J, Fan G, Ma L, Li S, Liu K (2024) Identification of a PET hydrolytic enzyme from the human gut microbiome unveils potential plastic biodegradation in human digestive tract. Int J Biol Macromol 283(Pt 3):137732. 10.1016/j.ijbiomac.2024.137732 39551294 10.1016/j.ijbiomac.2024.137732

[CR91] Zhao Y, Qin Z, Huang Z, Bao Z, Luo T, Jin Y (2021) Effects of polyethylene microplastics on the microbiome and metabolism in larval zebrafish. Environ Pollut 282:117039. 10.1016/j.envpol.2021.117039 33838439 10.1016/j.envpol.2021.117039

[CR92] Zhou H, Shi S, You Q, Zhang K, Chen Y, Zheng D, Sun J (2024) Polyethylene terephthalate hydrolases in human gut microbiota and their implications for human health. Microorganisms 12(1):138. 10.3390/microorganisms12010138 38257965 10.3390/microorganisms12010138 PMC10820491

